# How to avoid misinterpretation of dual reporter gene assay data affected by cell damage

**DOI:** 10.1007/s00204-022-03323-0

**Published:** 2022-06-09

**Authors:** Julie Nilles, Johanna Weiss, Walter E. Haefeli, Stephanie Ruez, Dirk Theile

**Affiliations:** 1 Department of Clinical Pharmacology and Pharmacoepidemiology, Heidelberg University Hospital, Im Neuenheimer Feld 410, 69120 Heidelberg, Germany; 2 Boehringer Ingelheim Pharma GmbH & Co. KG, Birkendorfer Str. 65, 88397 Biberach an der Riss, Germany

**Keywords:** Pregnane x receptor, Reporter gene assay, Cytotoxicity, Firefly, *Renilla*

## Abstract

The activity of nuclear receptors (e.g., pregnane x receptor, PXR) can be assessed by luminescence-based dual reporter gene assays. Under most conditions, receptor-activated firefly luminescence is normalized to *Renilla* luminescence, which is triggered by a constitutively active promoter. Simultaneous damage to the cells can however disrupt these signals and thus impair the interpretation of the data. Consequently, this study addressed three important aspects: First, idealized models were described, each highlighting crucial characteristics and important pitfalls of dual PXR reporter gene assays used to evaluate PXR activation or inhibition. Second, these models were supported by experimental data obtained with a strong PXR activator (rifampicin) with low cytotoxicity, a PXR activator with high cytotoxicity (dovitinib), a proposed PXR inhibitor that reportedly has no toxic effects (triptolide), and a cytotoxic control (oxaliplatin). Data were evaluated for relative PXR activity data, individual firefly or *Renilla* luminescence, and anti-proliferative effects of the compounds (assessed by crystal violet staining). Finally, a step-by-step guide is proposed to avoid misleading set-up of the assay or misinterpretation of the data obtained. Key considerations here include (1) omission of drug concentrations beyond 10–20% proliferation inhibition; (2) observation of *Renilla* luminescence, because this tends to indicate ‘false PXR activation’ when it inexplicably decreases; (3) parallel decrease of relative PXR activity and proliferation below baseline levels in conjunction with a sharp decrease in *Renilla* luminescence indicates ‘false PXR antagonism’; (4) non-sigmoidal relationships suggest the absence of concentration dependency.

## Introduction

There is great interest in characterizing drugs that activate or block signalling pathways that alter the expression or activity of genes that modulate pharmacokinetics. The pregnane x receptor (PXR) is a ligand-activated transcription factor that regulates the expression of the major drug-metabolizing cytochrome P-450 isozyme (CYP) 3A4 (Prakash et al. 2015; Pavek 2016). Its activation or inhibition can be estimated by numerous in vitro assays (e.g., scintillation proximity assay, fluorescence energy transfer, crystallography, surface plasmon resonance, fluorescence microscopy, etc.), but most of these methods are rather labour-intensive, indirect, or generate radioactive waste (Chai et al. 2019). In contrast, luminescence-based reporter gene assays directly reflect the transcriptional activity of nuclear receptors. For instance, PXR response elements of the promoter region of the CYP3A4 gene can be cloned upstream of the firefly luciferase-encoding gene (Gu et al. 2006). After transfection of cells with such a plasmid and adding the respective luciferase substrate, the activity of PXR is reported by enhanced firefly luminescence. Because the variable firefly luminescence could result from variable transfection efficiency, another reporter plasmid encoding a constitutively active *Renilla* luciferase is co-transfected. By dividing the firefly signals (indicating PXR activity) by the luminescence emitted by *Renilla* luciferase (reflecting transfection efficiency), transfection differences are accounted for in the result. The firefly/*Renilla* ratio consequently indicates the net activity of PXR.

In the experimental setting, the analysis of luminescence-based reporter gene assays can be difficult, because various modulators can influence the firefly/*Renilla* luminescence ratio and thus the interpretation of the data. For example, some drugs (e.g., anaesthetics) can attenuate firefly luminescence (Ueda et al. 1976; Dickinson et al. 1993; Keyaerts et al. 2012), some proteasome inhibitors protect firefly luciferase from degradation (Becker et al. 2016), and paclitaxel increases *Renilla* luminescence in a concentration-dependent manner (Theile et al. 2013). In addition, high drug concentrations used in the assay can cause cell damage (cytotoxicity, proliferation inhibition), which in turn can falsify the results, making it difficult to interpret the data. To date, there is no consensus on how to analyse or interpret luminescence-based reporter gene assays, especially when cell proliferation is simultaneously affected. Thus, we have developed a standard procedure to detect and minimise possible interfering influences in such experiments. We have developed idealized model scenarios with crucial characteristics and typical pitfalls of luminescence-based reporter gene assays. The models were subsequently verified by original data from PXR reporter gene assays with PXR activators without (rifampicin) or with (dovitinib) considerable anti-proliferative effects. To also cover the case of PXR inhibitors, triptolide was evaluated. This diterpenoide was recently proposed to be devoid of cell-damaging properties while efficiently inhibiting PXR activation (Zheng et al. 2021). Because our results clearly contradicted those findings, control experiments with oxaliplatin were performed. All data were subsequently depicted and discussed in regard to observed proliferation, relative PXR activity, and respective single firefly or *Renilla* luminescence signals because the latter has been suggested to be a very sensitive marker of cell damage (Lungu-Mitea and Lundqvist 2020). Having obtained these insights, a step-by-step instruction is proposed to prevent misleading assay setup or false interpretation of reporter gene assay data.

## Materials and methods

### Materials

Dulbecco’s Modified Eagle’s Medium (DMEM) and fetal calf serum (FCS) were purchased from PAN-Biotech (Aidenbach, Germany). Phosphate-buffered saline (PBS) and medium supplements (glutamine, non-essential amino acids, penicillin/streptomycin) were purchased from Sigma-Aldrich (Taufkirchen, Germany). Rifampicin, crystal violet, and dimethylsulfoxide (DMSO) were purchased from Applichem (Darmstadt, Germany) and methanol from Roth (Karlsruhe, Germany). Dovitinib was provided by Sequoia Research Products (Pangbourne, UK). Triptolide was purchased from Santa Cruz Biotechnology (Heidelberg, Germany). Oxaliplatin (dissolved in distilled water) was supplied by the University Hospital’s pharmacy. The Dual-Glo Luciferase Assay System, the pGL4.21 vector, the pGL4.74 [hRluc/TK] *Renilla* vector, and the FuGene® HD Transfection reagent were purchased from Promega Corporation (Madison, WI, USA). The *NR1I2* (NM_003889) human cDNA TrueClone® (pCMV6-XL4 vector, containing the cDNA of the PXR gene *NR1I2*) was obtained from OriGene (Rockville, MD, USA). Cell culture flasks and white 96-well plates with a white bottom (especially well-suited for luminescence measurements) were obtained from Greiner (Frickenhausen, Germany). The luminescence signal was detected with the SpectraMax iD3 from Molecular Devices (Wokingham, UK).

### Stock solutions

Rifampicin, dovitinib (100 mM stock solution), or triptolide (10 mM stock solution) were dissolved in DMSO. The stock solutions were freshly diluted with supplemented medium prior to the experiments. The DMSO concentrations in the assays did not exceed 0.1%. Oxaliplatin solution was diluted in double distilled water to obtain a 1 mM stock concentration. Subsequently, this stock was freshly diluted with supplemented medium prior to the experiments.

### Cell line and culture conditions

LS180 cells, a human colon adenocarcinoma cell line (available at ATCC, Manassas, VA, USA), were used for the experiments. This cell line is a well-established model for the PXR-driven induction of genes involved in the metabolism of xenobiotics (Harmsen et al. 2008; Gupta et al. 2008; Weiss et al. 2013). Cells were cultured under standard conditions with DMEM supplemented with 10% FCS, 2 mM glutamine, 100 U/mL penicillin, 100 µg/mL streptomycin sulphate, and 0.1 mM non-essential amino acids.

### Growth inhibition assay

The details of the assay have been published previously (Peters et al. 2006). Briefly, 50,000 LS180 cells per well were seeded and allowed to attach and grow overnight. For the evaluation of anti-proliferative effects during PXR activation assays, cells were treated with rifampicin or dovitinib. To evaluate anti-proliferative combination effects during the PXR inhibition assays, the cells were treated with the drug combinations rifampicin (5 µM) / triptolide or rifampicin (5 µM)/oxaliplatin. Cells were exposed to compounds of interest for 24 h at 37 °C with 5% CO_2_. Then the medium was removed and wells were washed with PBS and exposed to 50 µL crystal violet (0.5% in methanol) for 15 min on a rotary shaker. After removing the unbound crystal violet dye, wells were washed thrice with water to remove residual crystal violet (background reduction). After drying, the cell-bound crystal violet was dissolved in 200 µL/well methanol and detected at a wavelength of 555 nm. Each experiment was performed in three independent experimental replicates with *n* = 8 wells for each concentration/replicate. To calculate anti-proliferative effects, the mean values of the background absorbance were subtracted from the measured absorbance values of the samples and the untreated cell control was set to 100%. Concentration–response curves with a variable slope were calculated using GraphPad Prism version 9.1 (GraphPad Software Inc., La Jolla, CA, USA) according to a sigmoidal *E*_max_ model.

### Dual-luciferase reporter gene assay

#### Transfection of LS180 cells

The construction and basic principle of the PXR reporter gene vector have been described and published previously (Weiss et al. 2013). Briefly, the proximal response element module (PREM, comprising −362/+53 region) and the xenobiotic response element module (XREM, comprising −7836/−7208 region) of the CYP3A4 promoter had been cloned upstream of the firefly open reading frame of the pGL4.21 vector (Promega, Mannheim, Germany). The pGL4.74 [hRluc/TK] *Renilla* vector was used as a normalization vector to control for transfection efficiency. To ensure high expression of PXR, cells were co-transfected with pCMV6-XL4 containing the cDNA of human PXR (*NR1I2*).

For transfection, 50,000 cells per well were seeded and allowed to attach and grow overnight. The next day, the medium was replaced by a medium without supplements. Four hours later, transfection was performed with the lipid-based transfection reagent FuGene®. The ratio of transfection reagent to DNA was 5:1. Each well was exposed to 20 ng of the PXR expression vector, 80 ng of the reporter vector, and 10 ng of the Renilla vector. After the addition of the transfection reagent-DNA mix, the plate was shaken for 30 s at room temperature. Cells were then incubated at 37 °C for 24 h.

#### Measuring PXR activity/inhibition

For the PXR activity reporter gene assays, cells were treated with rifampicin or dovitinib for 24 h at 37 °C. In the PXR inhibition assays (triptolide or oxaliplatin, respectively), cells were initially pre-incubated with the proposed inhibitor for 60 min. Afterwards, the inhibitor was removed and a combination of rifampicin (5 µM) and the respective inhibitor was added to the cells for 24 h. Cells treated with 5 µM rifampicin only served as PXR activation controls. After treatment, recording of luminescence was performed using the Dual-Glo Luciferase assay system according to the manufacturer’s instructions with minor changes to the original protocol. The drug-containing medium was removed and replaced by a 40 µL drug-free cell culture medium. Then, 40 µL of firefly substrate-containing lysis buffer (luciferin) was added to the medium. The plate was incubated for 15 min on a rotary shaker at room temperature. After cell lysis, firefly luminescence was recorded using a luminometer (SpectraMax iD3). After detection of the firefly luminescence, 40 µL of the Stop&Glo reagent (containing the *Renilla* substrate coelenterazine) was added. The plates were again incubated for 15 min on a rotary shaker at room temperature and *Renilla* luminescence was also recorded.

PXR activity was calculated by dividing the firefly luminescence by the *Renilla* luminescence. Subsequently, obtained values were normalized to the mean value of the untreated control (set to 1). Percent firefly and *Renilla* signal alterations were calculated accordingly, setting the untreated cells to 100%. Each experiment was performed in three to six independent biological replicates with *n* = 4 wells for each concentration/replicate.

### Statistics

The impact of drug treatments on cell proliferation, relative PXR activity, or single luminescence values was evaluated by ANOVA with non-parametric Kruskal–Wallis test and Dunn’s test (controlling for multiple testing) using InStat Version 3.06 (GraphPad Software, San Diego, CA, USA). The impact of 5 µM rifampicin on relative PXR activity (compared to untreated control) and IC50 values of firefly and renilla luminescences (oxaliplatin as cytotoxic control) were evaluated by student’s *T* test using InStat Version 3.06. Concentration–response curves were plotted with GraphPad Prism version 9.1 (GraphPad Software Inc., La Jolla, CA, USA) according to a sigmoidal *E*_max_ model (four parameter-logistic equation; variable slope). A *P* value < 0.05 was considered significant.

## Results

### PXR activator with low cytotoxicity

#### Idealized model

**Fig. 1:**
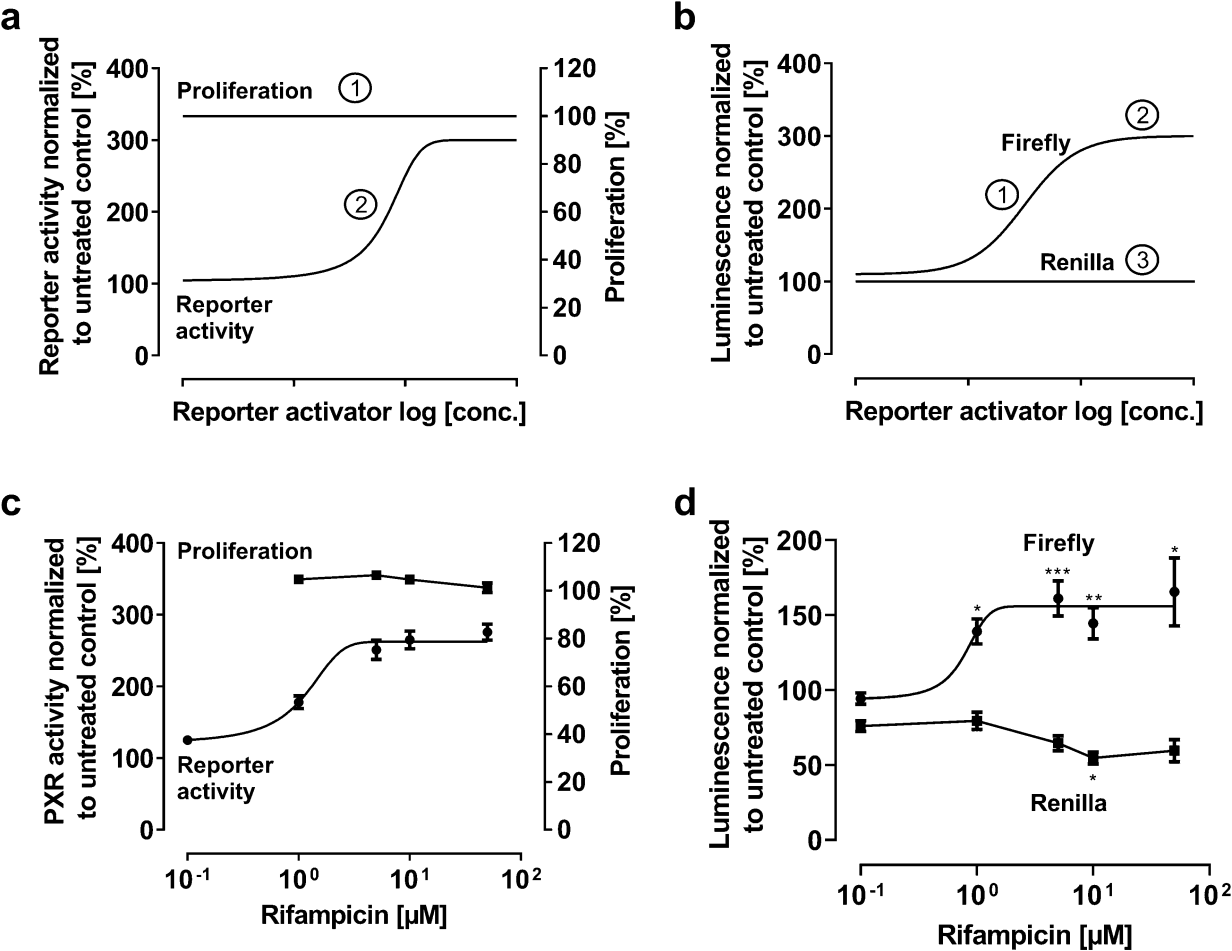
Relative PXR reporter activity and firefly or *Renilla* luminescence with an PXR activator exhibiting low cytotoxicity. Idealized model (upper panels): **a** ① Cell proliferation remains at 100%, ② Relative PXR activity shows a sigmoidal concentration-dependent increase; **b** ① The firefly luminescence increases in a sigmoidal manner and ② reaches a maximum, resulting in a plateau of relative PXR activity given ③ the constant *Renilla* luminescence. Experimental data (lower panels): **c** Rifampicin effect on cell proliferation and relative PXR activity after 24 h drug exposure, normalized to untreated control. **d** Firefly and *Renilla* luminescence normalized to untreated control. Data shown are the mean ± SEM of three independent biological replicates with *n* = 4 (reporter data) or *n* = 8 (proliferation data) replicates for each concentration/replicate. Whenever data could not be fitted to a sigmoidal Emax model (four parameter-logistic equation; variable slope), data points are simply connected (here: rifampicin effect on proliferation or *Renilla* luminescence). Impact of drug treatments on firefly or *Renilla* values was evaluated by ANOVA with non-parametric Kruskal–Wallis test and Dunn’s test compared to untreated control. *P < 0.05; **P < 0.01; ***P < 0.001

**Fig. 2:**
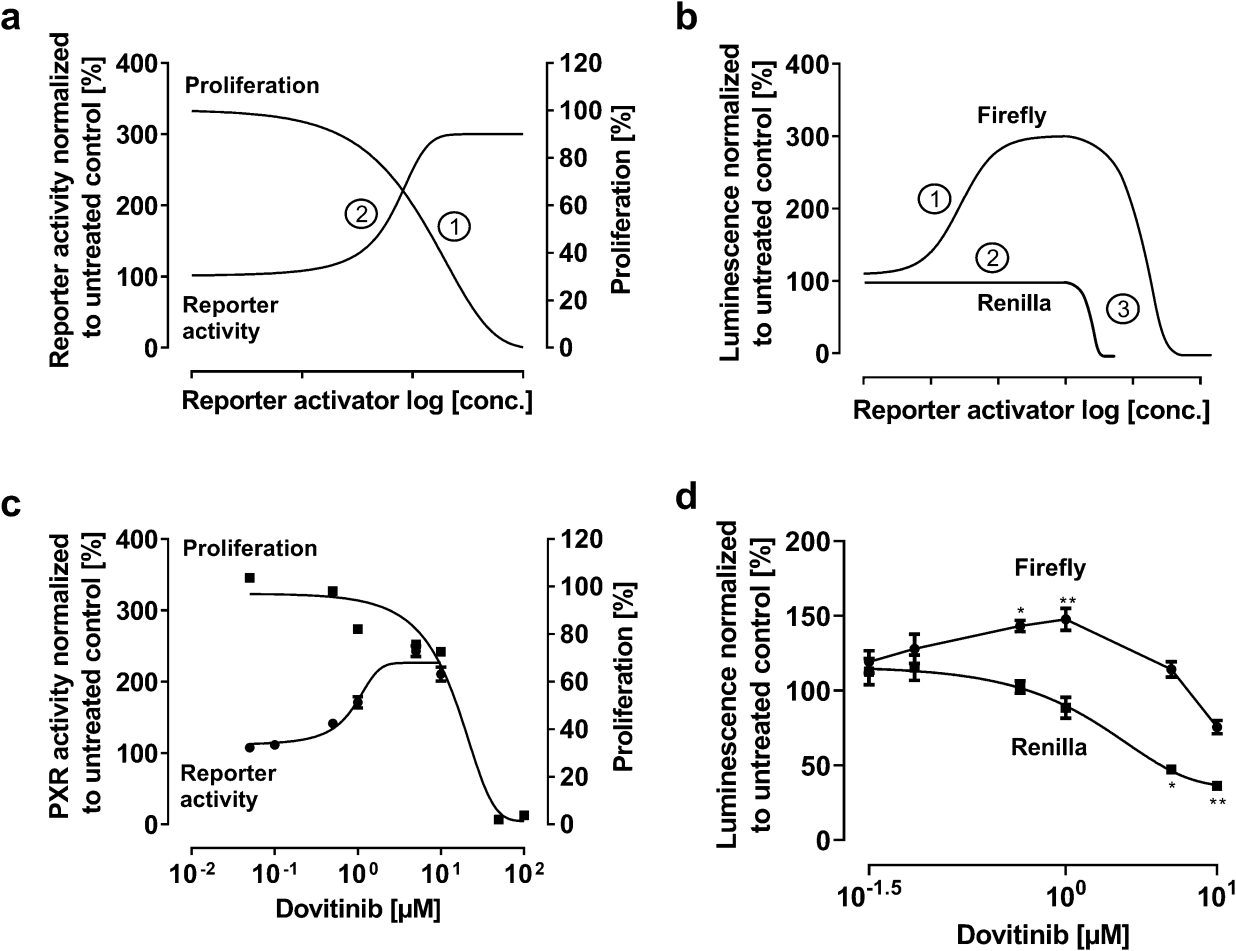
Relative PXR reporter activity and firefly or *Renilla* luminescence with an PXR activator exhibiting high cytotoxicity. Idealized model (upper panels): **a** ① Proliferation is decreased concentration-dependently, ② Relative PXR activity seems well enhanced by the drug of interest, reaching a certain maximum effect. **b** ① Initial sigmoidal increase of the firefly luminescence due to reporter activation. ② *Renilla* luminescence remains initially constant. ③ However, the parallel decrease of the firefly and *Renilla* luminescence maintain the impression of a relative PXR activity plateau. Experimental data (lower panels): **c** Dovitinib effect on cell proliferation and relative PXR activity after 24 h drug exposure, normalized to untreated control. **d** Firefly and *Renilla* luminescence normalized to untreated control. Data shown are the mean ± SEM of three independent biological replicates with *n* = 4 (reporter data) or *n* = 8 (proliferation data) replicates for each concentration/replicate. Whenever data could not be fitted to a sigmoidal Emax model (four parameter-logistic equation; variable slope), data points are simply connected (here: dovitinib effect on firefly luminescence). Impact of drug treatments on firefly or *Renilla* values was evaluated by ANOVA with non-parametric Kruskal–Wallis test and Dunn’s test compared to untreated control. *P < 0.05; **P < 0.01

**Fig. 3:**
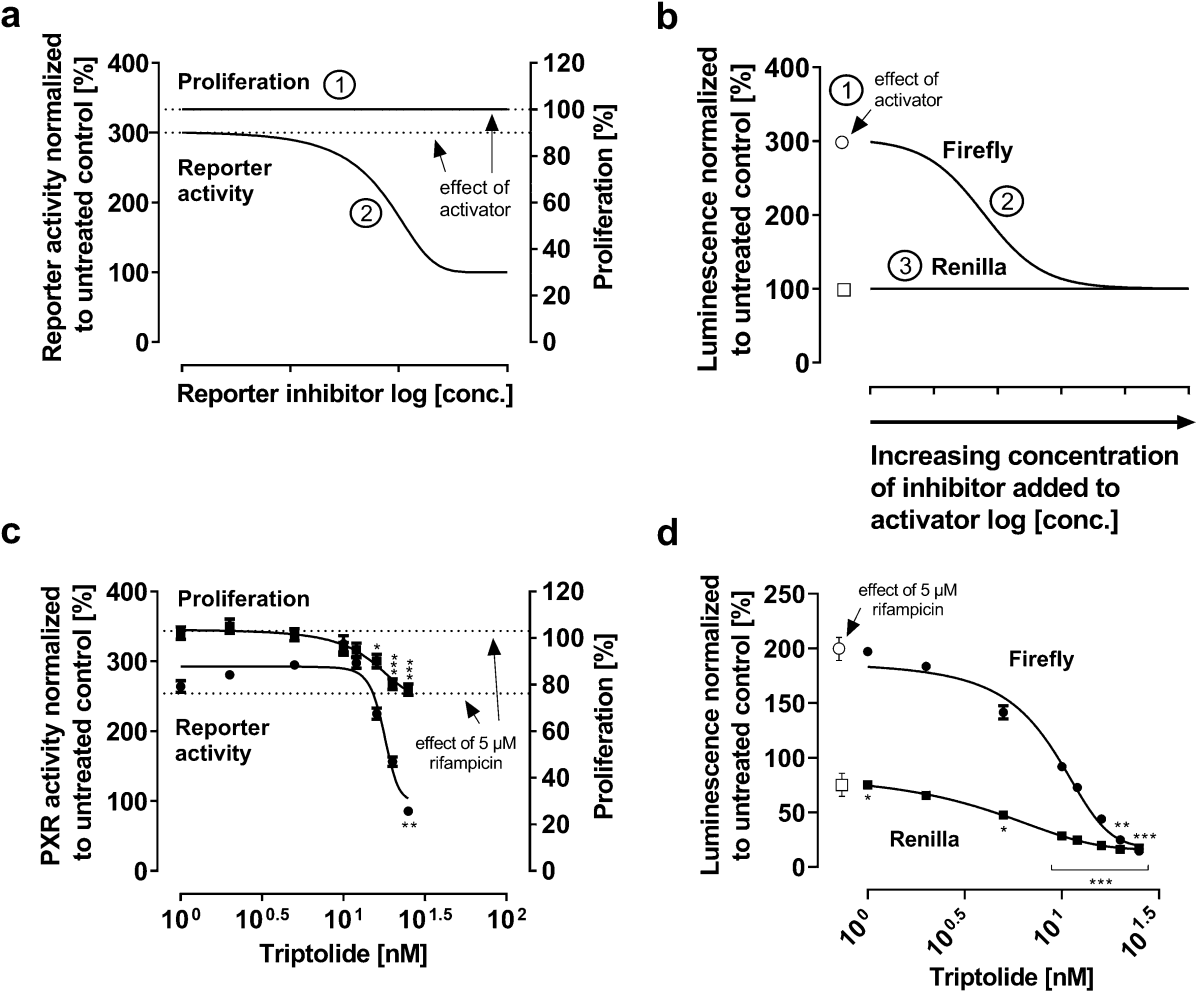
Relative PXR reporter activity and firefly or *Renilla* luminescence with a PXR inhibitor exhibiting low cytotoxicity. Idealized model (upper panels): **a** ① Proliferation is neither affected by the activator alone (e.g., rifampicin) nor by the proposed PXR inhibitor being added to the activator; ② PXR ligand-mediated enhancement of relative PXR activity (e.g. threefold compared to untreated control, dashed line) is concentration-dependently abolished by proposed PXR inhibitor being added to the activator. Eventually, relative PXR activity returns to the baseline level. **b** ① Strong PXR activator-mediated increase of the firefly luminescence (white circle), but unchanged Renilla signal (white square); ② Addition of an inhibitor leads to a sigmoidal decrease of the firefly luminescence; ③ *Renilla* luminescence remains constant. Experimental data (lower panels): **c** Impact of triptolide on cell proliferation and relative PXR activity when added to 5 µM rifampicin (24 h drug exposure). **d** Firefly and *Renilla* luminescence normalized to untreated control. Data shown is the mean ± SEM of three independent biological replicates with *n* = 4 (reporter data) or *n* = 8 (proliferation data) replicates for each concentration/replicate. Impact of drug treatments on firefly or *Renilla* values was evaluated by ANOVA with non-parametric Kruskal–Wallis test and Dunn’s test compared to untreated control. *P < 0.05, **P < 0.01, ***P < 0.001

PXR activators with low cytotoxic effects are characterized by 100% cell proliferation over the entire concentration range (Fig. 1a, ①). PXR activity is expected to increase concentration-dependently ultimately reaching a plateau (maximum efficacy, *E*_max_) (Fig. 1a, ②). Firefly luminescence is also expected to increase to a plateau in response to PXR activation (Fig. 1b, ①, ②). In contrast, Renilla luminescence is expected to remain constant over the entire concentration range of the activator (Fig. 1b, ③).

### Experimental data: Rifampicin

In the concentration range evaluated, rifampicin had no anti-proliferative effects (for all concentrations *P* > 0.05) and increased relative PXR activity in a concentration-dependent manner, reaching a maximum increase of PXR activity of 2.7-fold ± 0.2 at 5 µM rifampicin (*P* = 0.0004 compared to untreated control cells). Concurrently, the firefly and *Renilla* luminescence values resembled the idealized model. After a considerable initial sigmoidal increase of firefly luminescence, a maximum is reached at 5 µM rifampicin (10, 50, 100 µM non-significantly different from 5 µM). In contrast, *Renilla* luminescence remained stable without obvious concentration dependency (Fig. 1d), hindering a fitted sigmoidal model of the *Renilla* data.

### PXR activator with high cytotoxicity

#### Idealized model

Cytotoxic reporter gene activators should cause a concentration-dependent decrease of proliferation (Fig. 2a, ①). Initially, there is an increase in relative PXR activity (Fig. 2a, ②), because firefly luminescence increases while *Renilla* luminescence remains constant (Fig. 2b, ①, ②). However, at a certain concentration, profound cytotoxicity will cause a parallel decrease of both signals (= constant ratio), mimicking a plateau of relative PXR activity (Fig. 2b, ③).

### Experimental data: Dovitinib

Dovitinib inhibited cell proliferation concentration dependently (Fig. 2c). Despite anti-proliferative effects, there was an (non-sigmoidal) increase in firefly luminescence (Fig. 2d), resulting in an increase in relative PXR activity (Fig. 2c). However, this increase was additionally amplified by the sigmoidal decrease of the *Renilla* signal (Fig. 2d). At 1 µM, both luminescence values started to decrease in parallel (= constant ratio) (Fig. 2d), a concentration where relative PXR activity seemed to plateau accordingly (Fig. 2c).

### PXR inhibitor with low cytotoxicity

#### Idealized model

A PXR inhibitor with low cytotoxicity is supposed to not affect cell proliferation (Fig. 3a, ①). At low concentrations of the inhibitor, PXR activation (e.g., rifampicin-mediated threefold increase, dashed line) is expected to prevail, whereas there will be a sigmoidal decrease of reporter gene activity with higher concentrations of the inhibitor added to the activator (Fig. 3a, ②). A maximum inhibition, relative PXR activity is decreased back to the baseline of untreated cells (set to 100%) (Fig. 3a, ②). Regarding the single luminescence data, the firefly luminescence is expected to be strongly enhanced by the activator (Fig. 3b, ①), whereas it steadily decreases in a sigmoidal manner by the co-treatment with an inhibitor (Fig. 3b, ②). Given the null effect on cell proliferation, the *Renilla* signal is expected to remain constant (Fig. 3b, ③).

### Experimental data: Triptolide

Rifampicin at 5 µM did not affect cell proliferation (Fig. 3c; dashed line at 100% proliferation). Relative PXR activity was enhanced 2.5-fold ± 0.1 (*P* < 0.0001) compared to untreated cells (Fig. 3c; dashed line of PXR activity). However, adding triptolide decreased cell proliferation (>16 nM, *P* < 0.05) and rifampicin-mediated PXR activation (>25 nM, *P* < 0.01) (Fig. 3c). Concentrations beyond 20 nM (*P* < 0.01; *P* < 0.001 for 25 nM) caused a sigmoidal decline of firefly luminescence below baseline. In addition, *Renilla* luminescence also dropped below baseline in a sigmoidal manner (5 nM, *P* < 0.05; all other concentrations *P* < 0.001), suggesting considerable cell damage and eventually contradicting the idealized model described above.

### PXR inhibitor with high cytotoxicity

#### Idealized model

**Fig. 4:**
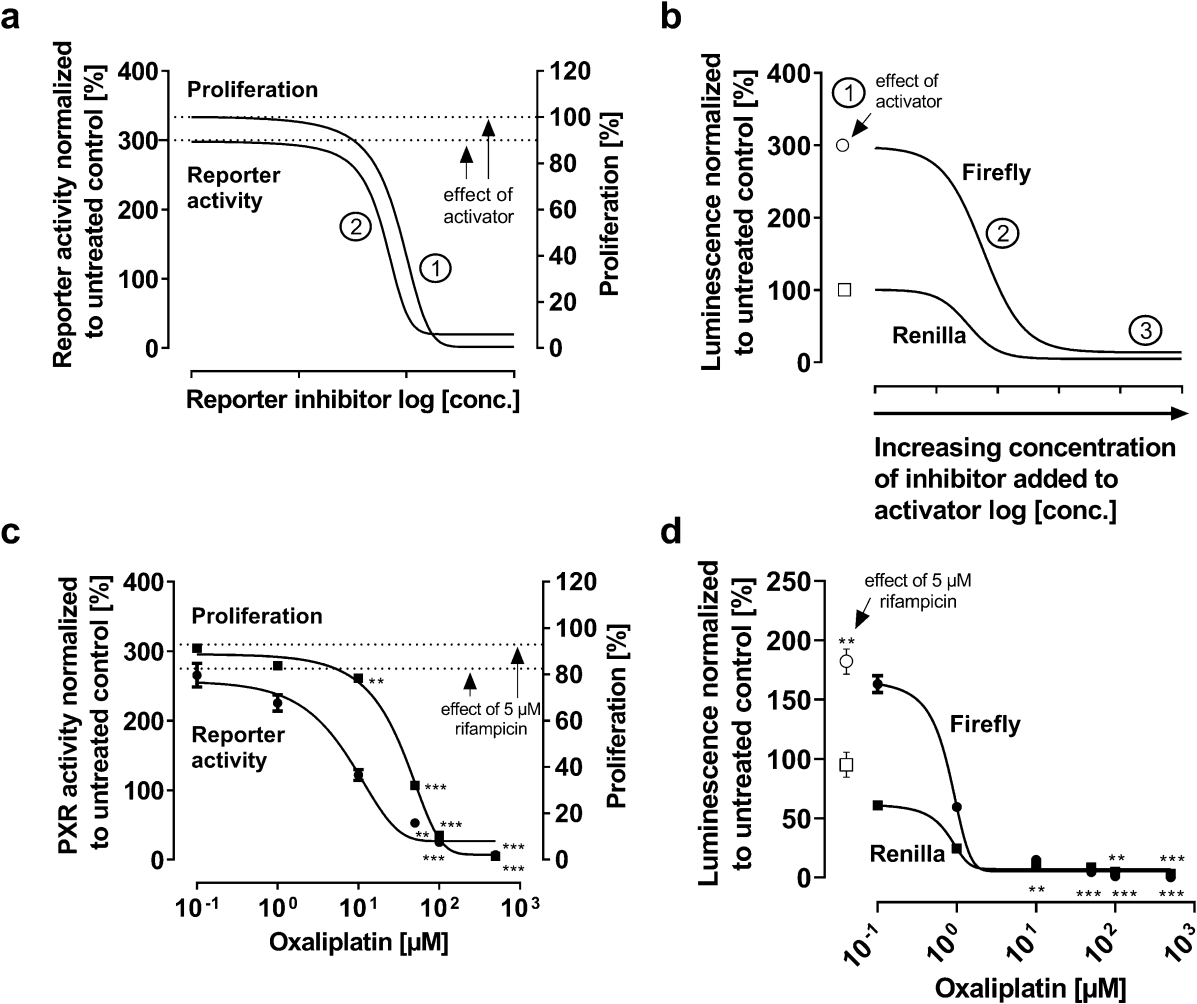
Relative PXR reporter activity and firefly or *Renilla* luminescence with a PXR inhibitor exhibiting high cytotoxicity (idealized model) and relative PXR reporter activity and firefly or *Renilla* luminescence of oxaliplatin as a cytotoxic positive control (experimental data). Idealized model (upper panels): **a** ① Proliferation is not affected by the activator (e.g. rifampicin). But adding the cytotoxic compound decreases cell proliferation concentration-dependently; ② PXR ligand-mediated enhancement of relative PXR activity (e.g. threefold compared to untreated control) is concentration-dependently decreased below baseline levels by the cytotoxic drug. **b** ① Strong PXR activator-mediated increase of the firefly luminescence (white circle) but unchanged *Renilla* signal (white square); ② Addition of the cytotoxic compound leads to a sigmoidal decrease of both the firefly and *Renilla* luminescence; ③ The signals converge at a very low luminescence level. Experimental data (lower panels): **c** Impact of oxaliplatin on cell proliferation and relative PXR activity when added to 5 µM rifampicin (24 h drug exposure). **d** Firefly and *Renilla* luminescence normalized to untreated control. Data shown is the mean ± SEM of three-six independent biological replicates with *n* = 4 (reporter data) or *n* = 8 (proliferation data) replicates for each concentration/replicate. Impact of drug treatments on firefly or *Renilla* values was evaluated by ANOVA with non-parametric Kruskal–Wallis test and Dunn’s test compared to untreated control. A *P* value < 0.05 was considered significant. **P < 0.01, ***P < 0.001

Because triptolide was rather anti-proliferative, the impact of a potent cytotoxic drug on PXR activation was described. Again, the PXR activator alone is expected to not affect proliferation (Fig. 4a; dashed line at 100% proliferation) while enhancing relative PXR activity (e.g., threefold enhancement, dashed line of PXR activity, Fig. 4a). The addition of a cytotoxic drug will decrease cell proliferation and, concurrently, directly inhibit PXR activation (Fig. 4a, ①). Noteworthy, relative PXR activity decreases in parallel to proliferation and ends below the PXR activity level of untreated cells (Fig. 3a, ②). Regarding single luminescence data, the PXR activator alone (e.g. rifampicin) is expected to enhance firefly luminescence (e.g. threefold compared to untreated control) without impact on *Renilla* luminescence (Fig. 4b, ①). An added cytotoxic compound will cause a sigmoidal and parallel decrease of firefly luminescence and *Renilla* luminescence. Eventually, both luminescence values are expected to converge to a very low signal detection level well below baseline levels because very few cells survived the treatment and emit luminescence.

### Experimental data (oxaliplatin, a cytotoxic control)

To assess how luminescence signals are affected by cytotoxicity, a series of experiments with oxaliplatin was performed. Rifampicin at 5 µM again had no impact on cell proliferation (Fig. 4c; dashed line at about 100% proliferation) but enhanced PXR activity 2.8-fold ± 0.4 (*P* = 0.0018) compared to untreated cells (Fig. 4c; dashed line of PXR activity). Addition of oxaliplatin concurrently decreased cell proliferation (10 µM, *P* < 0.01;  >50 µM, *P* < 0.001) and relative PXR activity (50 µM, *P* < 0.01; >100 µM, *P* < 0.001) in a sigmoidal manner, ultimately reaching PXR activity levels below baseline (100 µM, *P* < 0.05; 500 µM, *P* < 0.001) (Fig. 4c), suggesting that ‘PXR inhibition’ had been mimicked by cell damage. Accordingly, firefly (100 µM, *P* < 0.01; 500 µM, *P* < 0.001) and *Renilla* (10 µM, *P* < 0.01; >50 µM, *P* < 0.001) luminescence signals were decreased in a sigmoidal manner below baseline level. Interestingly, IC50 values for luminescence declines were the same for firefly (0.8 ± 0.04 µM) and *Renilla* (0.8 ± 0.01 µM; *P* = 0.6675).

## Discussion

During the pre-clinical phase of drug development, the risk of a given drug to cause pharmacokinetic drug–drug interactions is routinely assessed. Because PXR reporter gene assays can be an important part of this investigation (Jones et al. 2017), the methodology needs to be correctly established, exactly executed, and its results thoroughly interpreted. Dual PXR reporter gene assays rely on the normalization of reporter signals from the firefly luciferase to signals emitted from the *Renilla* control vector. Consequently, off-target effects on the single luminescence values can lead to ‘false PXR activation’ or ‘false PXR antagonism’. Because cytotoxic or anti-proliferative effects are amongst the most common confounders in experimental research, this study addressed the relationship between anti-proliferative effects and the single firefly or *Renilla* signals, being read-outs for relative PXR activity.

Rifampicin was used as a proto-typical PXR activator (Nakajima et al. 2011; Li et al. 1997; Chen and Raymond 2006) and the data verified the idealized model of an activator without anti-proliferative effects. Firefly luminescence increased until a certain maximum, while *Renilla* luminescence remained largely constant, leading to a net increase of relative PXR activity (firefly/*Renilla* ratio) and an obvious plateau. In contrast, dovitinib was used as an anti-proliferative compound (Ma et al. 2019) with an unclear impact on PXR (Weiss et al. 2014). The current findings (Fig. 2c) suggested some degree of PXR activation but this was at least in part mimicked by the divergence of firefly and *Renilla* luminescence. For instance, at 0.1–1 µM dovitinib, there is a decrease in *Renilla* luminescence, which contributes to the apparent enhancement of relative PXR activity (Fig. 2c). At high, anti-proliferative concentrations of dovitinib (>1 µM), firefly and *Renilla* luminescence started to decrease in parallel (= constant ratio), maintaining a plateau of elevated PXR activity. Noteworthy, the firefly dynamics did not follow a sigmoidal course of concentration dependency, again suggesting that this PXR activation was concurrently overlapped by cell damage.

Recently, triptolide was proposed to selectively repress the transcriptional activation of human PXR by 10 µM rifampicin with only little cytotoxic effects (Zheng et al. 2021). However, a relevant weakness of that data was that the inhibitory effect was presented as a percentage decrease of PXR activity, normalized to 10 µM rifampicin but not normalized to untreated cells. In consequence, the total effect size of triptolide could not be evaluated. Moreover, it is not clear whether the decrease in PXR activity had been due to genuine PXR inhibition or caused by cytotoxic effects. In our data, proliferation concurrently decreased with relative PXR activity, suggesting an overlap of PXR inhibition and anti-proliferative effects. This is underlined by the sharp sigmoidal drop of both firefly and *Renilla* luminescence well below the baseline level (Fig. 3d). So far, other compounds proposed to be potent PXR inhibitors all have high anti-proliferative properties as well, being in line with their inherent mode of action, e.g. camptothecin (topoisomerase inhibitor; Chen et al. 2010), pimecrolimus (calcineurin inhibitor), or pazopanib (tyrosine kinase inhibitor; Burk et al. 2018). We are not aware of a PXR inhibitor devoid of cytotoxic or anti-proliferative effects and it appears that whenever considerable PXR inhibition was hypothesized so far, cell damage likely influenced the findings.

To verify this assumption and to benchmark the data on triptolide, experiments with a cytotoxic positive control were conducted. The data on oxaliplatin clearly confirmed that toxic compounds can cause parallelism of proliferation and relative PXR activity. Again, the relative PXR activity suspiciously dropped below the level of the untreated cells. That means the firefly/*Renilla* ratio of oxaliplatin-treated cells was lower than the ratio of untreated cells. This occurs when both luminescence values of the treated cells approach zero (dead cells do not emit), leading to a firefly/*Renilla* ratio close to 1, being definitely lower than the firefly/*Renilla* ratio of untreated cells (firefly, approx. 50,000 light units; *Renilla*, approx. 3000 light units). In summary, the control experiments with oxaliplatin confirmed that parallelism of proliferation with relative PXR activity or merging firefly and *Renilla* luminescence values (at a very low emission level) are highly suspicious and should alert any experimenter.

Limitations of our study need to be mentioned: The proliferation assays using the crystal violet staining method were performed with non-transfected cells, although previous studies had shown that the transfection manoeuvre itself can cause cell damage (Lungu-Mitea and Lundqvist 2020). However, this had become most apparent with very high drug concentrations used in the dimethylthiazole-carboxymethoxyphenyl-sulfophenyl-tetrazolium (MTS) assay. While the MTS assay is a popular method to assess cell viability, its read-out (NADPH turnover) is influenced by many cellular (e.g., alternative signalling pathways) or experimental (e.g., cell culture supplements) confounders (Stepanenko and Dmitrenko 2015). In contrast, crystal violet staining is an easy-to-perform method that simply indicates the abundance of cells. In consequence, this assay concurrently indicates cytotoxicity (removal of dead cells during the washing steps) and proliferation inhibition (unaffected cells divide, leading to higher staining intensity). Finally, the data presented here refer to a PXR reporter gene assay only and cannot uncritically be generalized to any other signalling pathway. However, because luciferase-based reporter assays are very common and the emitted luminescence eventually results from the luciferase protein (not the promoter upstream of the encoding sequence), it seems prudent to mind this data also for other luminescence-based reporter gene assays.

## Conclusions

To obtain reliable dual reporter gene data, we recommend the following: First, growth inhibition assays in a large concentration range should help establish IC_10_ concentrations as a maximum concentration for subsequent reporter activation experiments (reporter activator only). Second, growth inhibition assays and reporter gene assays should be performed with the same drug concentrations or drug combinations used in the reporter antagonism studies (reporter activator + reporter inhibitor). Third, data on proliferation and relative reporter gene activity should be depicted in the same graph. This allows for alignment with the idealized models presented here and thus estimation of the mode of action (e.g. genuine PXR activation, ‘false PXR inhibition’, etc.). Fourth, the firefly and *Renilla* data should be analyzed separately but depicted in the same graph, again allowing for recognition of mimicked relative PXR effects. Fifth, sigmoidal concentration–response curves of signals advocate for a true drug effect (e.g. sigmoidal firefly increase by activators; sigmoidal *Renilla* decrease by toxic effects), whereas non-sigmoidal relationships suggest the absence of concentration dependency or overlapping effects. By following these instructions, the most suspicious findings can be tracked: (i) Relative reporter activity or firefly/*Renilla* signals clearly below baseline level (untreated cell control) suggest cell damage. (ii) A parallel decrease of proliferation and relative reporter activity likewise suggests cell damage and thus unreliable data. Taken together, whenever one of these phenomena is detected in the data, a ‘false’ reporter activation or inhibition should be assumed.
